# Designing and Delivering a Task-Based, Structured, Standardized Patient Encounter Session in Clinical Skills Courses

**DOI:** 10.7759/cureus.114650

**Published:** 2026-08-17

**Authors:** Ghaith Al-Eyd, Thura Al-Khayat, Anita Laloo, Gary Schwartz

**Affiliations:** 1 Department of Medical Education, Nova Southeastern University Dr. Kiran C. Patel College of Allopathic Medicine, Fort Lauderdale, USA; 2 Department of Obstetrics and Gynaecology, Arrowhead Regional Medical Center, Colton, USA

**Keywords:** clinical reasoning, clinical skills, curriculum integration, sp session, standardized patient encounter

## Abstract

Clinical skills (CS) are linchpins of medical school curricula, integrated across all four years of training. Medical schools vary noticeably in how they deliver CS content, particularly during the pre-clerkship years, and much of that variability reflects the absence of a standardized structure for small group CS sessions. A well-defined session structure ensures consistent learning opportunities to practice the full range of required clinical reasoning-based tasks: hypothesis-driven history taking, physical examination, prioritized differential diagnosis formulation, diagnostic and initial therapeutic planning, encounter closure, and oral case presentation.

Given the inherent constraints of allotted session time and course duration, ensuring equitable student participation in a small group setting is a persistent challenge. To address this, we developed a structured approach to delivering standardized patient encounter-based CS content that allows regular practice of all assigned session tasks throughout the course. Upon introduction of the new method, students immediately recognized its positive impact; a sentiment that triggered adopting the method among faculty and then conducting a study to explore students’ insight into its effectiveness. The study included a five-point Likert scale survey, related open-ended questions, and statistical and content analyses.

The results showed high ratings (4.27/5 and 4.31/5), supported by positive sentiments on the impact on skills development, teamwork, and opportunities to practice a comprehensive range of CS. Our task-based, structured, standardized patient encounter method not only promotes equitable learning opportunities but could also enhance CS acquisition through basic clinical sciences integration and optimization of faculty capacity to deliver CS sessions and evaluate students’ learning effectively.

## Introduction

The ultimate goal of medical school curricula is to equip graduating medical students with the knowledge, skills, and values needed to become competent physicians. Clinical skills (CS) are the core elements of the educational process that shape students’ approach to patient care while they attain related competence. Although the term “clinical skills” implies a specific skill domain, its application in patient encounters integrates medical knowledge, communication, and interpersonal skills as cross-domain structured learning elements [1].

Multiple learning theories can be applied in medical education, including cognitivism, constructivism, experiential learning, adult learning, self-directed learning, community of practice and situated learning, cognitive apprenticeship, behaviorism, and reflective learning [2]. Medical students are adult learners who can observe and perform CS through self-directed learning enhanced by faculty skill demonstration and feedback, and application of most of these theories can be used to achieve the CS learning expectations [1,3].

CS faculty may apply various teaching methods to guide students’ CS learning based on the nature of the curriculum and course setting, including popular approaches such as Peyton’s and Robert Gagne's methods. Although these two methods vary in structure, the learning process in both is structured into core stages including skill demonstration, explanation, practice, and guiding feedback [4,5]. Based on these methods, various approaches have been designed to efficiently organize session delivery and effectively maximize both acquisition and application of knowledge and skills. These approaches have a learning process scaffolding based on core elements: cognitive skill fundamentals, sequenced skill demonstration by faculty and learners with and without step explanations, learner practice, and constructive faculty feedback [6-9]. Other common approaches incorporate aspects of the Objective Structured Clinical Examination in the CS learning process, including clinical reasoning exercises, oral presentation of case findings, note documentation, and formulation of clinical questions [10]. While CS teaching is primarily a faculty-led process, peer teaching can also indirectly enhance the CS learning process through observation of peers' practice in small groups or directly in formal peer-led teaching sessions [11]. 

As emphasized in all CS teaching approaches, learning CS requires not only observing and repeatedly practicing skills under guiding feedback, but also applying relevant medical knowledge into the reasoning process that guides skills applications in patient encounters [1,6-12]. Hence, the plan of an ideal CS learning session would include various components that address clinical reasoning exercises, hypothesis-driven history and physical examination, formulation of a prioritized differential diagnosis, discussion of an initial management plan, encounter closure, and oral presentation of the case findings. 

Since CS sessions are delivered as small group (six-eight students) sessions, a lack of a session structure that ensures equal participation opportunity would impact comparability of learning and practice among the students. To address this important learning variability factor and to cover all integral aspects of CS learning, we designed an inclusive multi-task structured CS session plan. In this plan, students rotate through different tasks during the weekly CS session to apply relevant medical knowledge into skills learning and practice. The objective of this study is to share our innovative approach as well as to evaluate students’ perceptions of its effectiveness and acceptability.

The innovative approach reported in this article was previously presented as a meeting abstract poster at the 2023 Director of Clinical Skills Education (DOCS) Annual Meeting on November 3, 2023, in Seattle, Washington, United States.

## Materials and methods

This study was conducted at Dr. Kiran C. Patel College of Allopathic Medicine, Nova Southeastern University, Florida, United States. The study protocol was reviewed and exempted by the Nova Southeastern University Institutional Review Board (protocol number: 2023-505).

The innovation

In our CS courses, faculty and course directors have met regularly to discuss the implementation of the course activities and explore potential improvements to the learning and assessment process. To optimize acquisition of knowledge and skills in these courses, course directors used assessment and evaluation results as outcome measures. Among these data were course evaluations by students and student evaluations of teaching effectiveness of individual faculty. Two CS course directors approached two of the CS faculty who had consistently been receiving outstanding evaluations from students, and discussed their individual approach to the weekly CS sessions, focusing on how they organized and delivered their sessions to ensure engagement of all students. After meeting with each of those two faculty members individually and discussing the description of their teaching philosophy and methods, the course directors recognized the need to explicitly structure the weekly Standardized Patient (SP) encounter CS sessions based on tasks that address different aspects of the encounter. Accordingly, and in collaboration with the two identified faculty members, the course directors created an inclusive session plan that incorporated combined core elements from the approaches those two faculty members used, which ensured engagement of each student in the group using a rotational pattern during sessions across the course.

The team formulated this session plan into a teaching method named “Task-Based Structured Standardized Patient Encounter” (TBS-SPE). The session plan was fully explained during the faculty course orientation meeting, and all faculty received an electronic version of the session plan structure that listed the students of their individual groups and showed the session steps and rotating tasks to be assigned to each student. Although the TBS-SPE was recommended to the course faculty, its implementation might have varied slightly in timing; however, the task assignment and the rotating schedule remained consistent.

**Figure 1:**
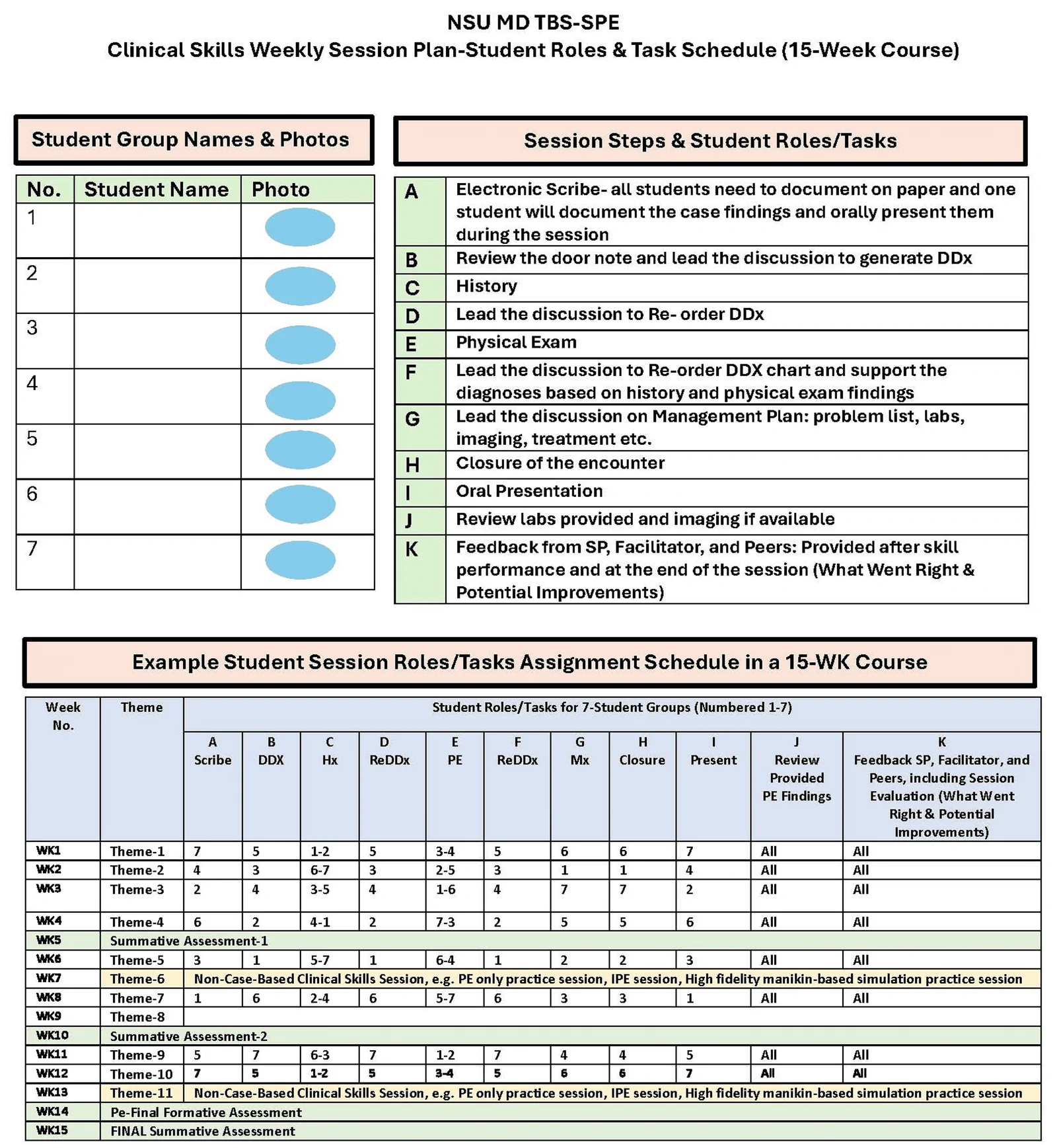
TBS-SPE Session Plan DDx: differential diagnosis; ReDDx: reorder DDx; PE: physical examination; Mx: management; TBS-SPE: Task-Based Structured Standardized Patient Encounter; NSU: Nova Southeastern University; WK: week; SP: standardized patient Image Credit: Authors; this figure is a snapshot image of the combined session guide documents developed as Microsoft Word tables (Microsoft Corporation, Redmond, Washington, United States) and then combined in one PDF document (Adobe Acrobat; Adobe Inc., San Jose, California, United States).

Figure 1 shows snapshots from the sections of the session plan shared with the faculty and recommended for the delivery of the weekly SP CS sessions. 

The session plan included: (i) Identifying different tasks of the session listed in the figure as “A-K”, (ii) Assigning numeric codes for students in the group (e.g. 1-7), and (iii) Creating a randomly rotating schedule that assigns two students for each task “C” (History Taking) and “E” (Physical Examination), and one student for the rest of tasks.

The sequenced schedule covered all the weekly SP encounters and ensured that each student participated multiple times in different tasks throughout the course. While only two students were primarily assigned to the physical examination task in each weekly session, the rest of the students would all practice that physical examination in the remaining session time after all tasks of the process were performed. Students would first generate a broad pre-encounter differential diagnosis based only on the door note findings that included the patient’s name, age, sex/gender, chief complaint, and vital signs. For each diagnosis identified, the students would list any related risk factors, discuss the possible underlying pathophysiology, and provide the expected history and physical examination findings. Students would revisit the broad list of differential diagnoses one time after collecting history findings and again after the physical examination to shortlist and reorder the diagnoses identified and finally formulate a prioritized list of three different diagnoses.

Feedback was provided on each student’s performance by the SP, peers, and faculty after history taking, physical examination, and encounter closure. Faculty also provided feedback on oral presentation and facilitated the student-led clinical reasoning discussion on the differential diagnosis and management plan.

End-of-course evaluations of students' perceived effectiveness

Since many students from different groups verbally shared how effective the TBS-SPE method was, the CS course directors made this method a recommended teaching approach for the weekly CS sessions, and two dedicated questions were added to the anonymous end-of-course student evaluations. Students were required to participate in this end-of-course evaluation, which was reviewed and validated by the Office of Curricular Affairs as part of the program evaluation.

One question was a five-point Likert scale type, and the other was an open-ended free-response question. The Likert scale question asked, “Regarding the clinical skills learning method of rotating task assignments during the weekly SP encounter sessions, please rate how effective this method was in enhancing skills acquisition”. The open-ended free-response question asked, “Regarding the question above, if you found the session effective, please explain in what way, e.g., enhances leadership, teamwork, etc.”

The data were collected from two cohorts of students in two CS courses, Practice of Medicine (POM) 2 and 3, in two successive academic years, 2024-2025 and 2025-2026, where POM-2 students are first-year (M1) medical students, and POM-3 students are second-year (M2) medical students.

Data analyses

The statistical data analyses done for the Likert scale question results included mean, median, standard deviation (SD), and a Wilcoxon non-parametric test, which was run to determine if there was a statistically significant difference (P < 0.05) in the distributions of the data between the study groups (POM-2 and POM-3 students). Statistical analysis was done using JMP® Pro 18.0.0 (JMP Statistical Discovery LLC, Cary, North Carolina, United States) and R 4.2.2 (R Foundation for Statistical Computing, Vienna, Austria). The data analyses for the open-ended free responses included prompting an artificial intelligence (AI) platforms to address “Thematic”, “Sentiment”, and “Content” analyses. Claude Sonnet 4.6 (Anthropic, PBC, San Francisco, California, United States) was able to specifically address all aspects of our prompts on analyzing common theme distribution among the responses, the sentiment category distribution, content analysis for word frequency distribution, and response length by word count. The sentimental categories were defined as “Strongly Positive” (enthusiastic endorsement of the rotating task format), “Mildly Positive” (positive acknowledgment with functional or descriptive language), “Neutral” (descriptive/very brief with no strong evaluative language), “Mildly Negative” (identifies problems, unmet expectations, or implementation gaps), and “Strongly Positive” (explicit reporting of negative impact of the approach on learning). The content analysis addressed the response length by word count (short <= 15, Medium =16-50, Long >50), word frequency, mean, and median. The results of all analyses were carefully reviewed by the authors, and the final sets of results were further developed through Microsoft Excel (Microsoft Corporation) based on data that were consistently provided by the AI platform.

## Results

The total number of students registered in both courses for the two academic years 2024-2025 and 2025-2026 was 225, and all students responded to the survey’s questions: 113 students from the POM-2 course and 112 students from the POM-3 course. 

Table 1 shows that the mean score for the five-point Likert scale question was 4.27 (SD 0.845) for POM-2 students and 4.31 (SD 0.921) for POM-3 students. Wilcoxon non-parametric test shows a p-value of 0.398, indicating no statistically significant difference between the scores of POM-2 and POM-3 students.

**Table 1:** Results of the Five-Point Likert Scale Survey Question The survey question was “Regarding the clinical skills learning method 'rotating task assignments during the weekly SP encounter sessions', please rate how effective this method was in enhancing skills acquisition”. P < 0.05 was considered significant. POM: Practice of Medicine

Total Number of Students/Course	POM 2 (N=113)	POM 3 (N=112)	P-value
Mean (SD)	4.27 (0.845)	4.31 (0.921)	0.398
Median (Range)	4.00 (1.00, 5.00)	5.00 (1.00, 5.00)	

Of the total 225 students, 72 (32%) answered the open-ended, free-response question that asked students to reflect on the effectiveness of rotating task assignments within the small-group CS sessions. The free responses were analyzed for sentiments, common themes, word frequency, and response length. 

**Figure 2:**
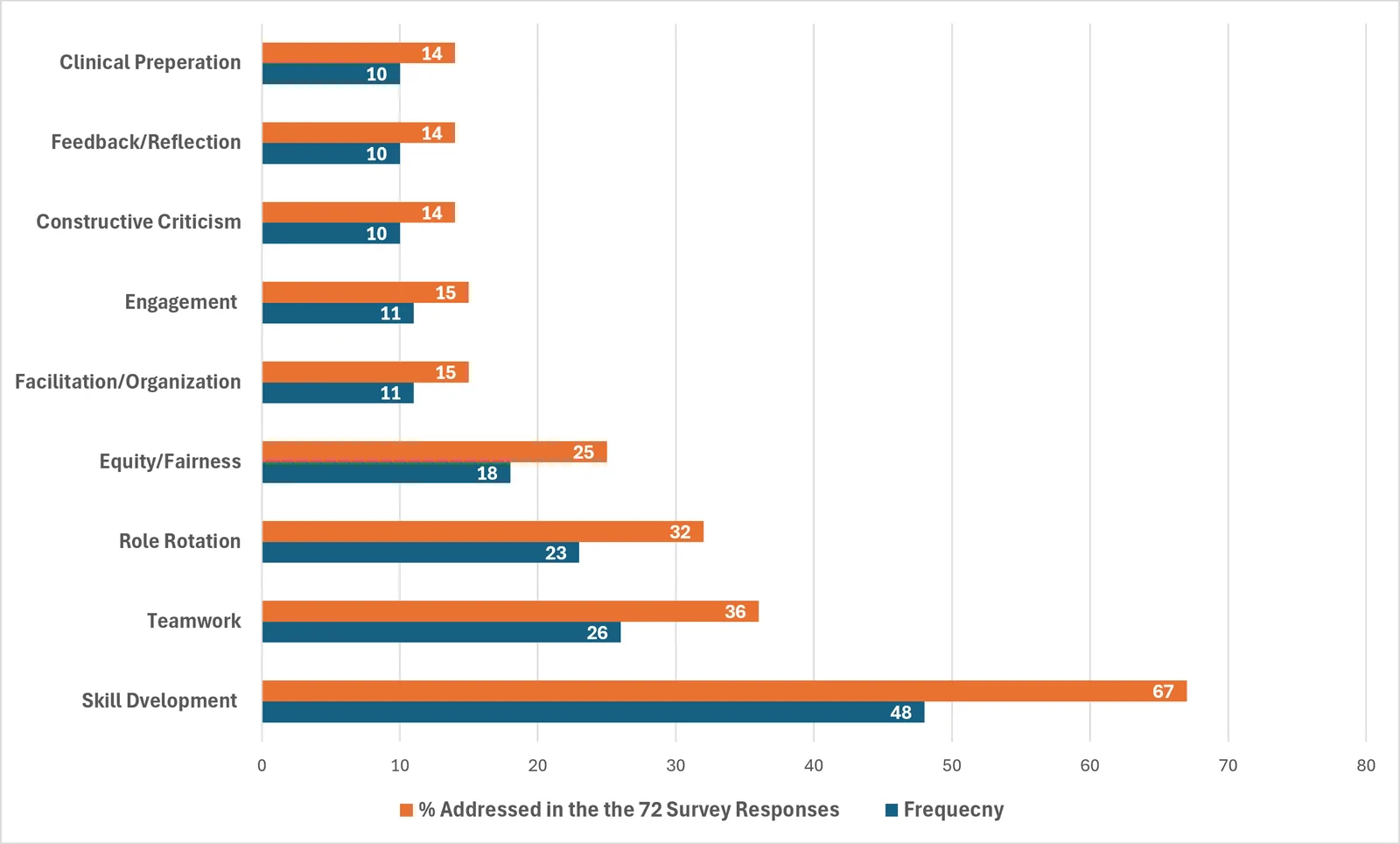
Distribution of the Open-Ended, Free Responses According to Themes

Figure 2 shows the thematic analysis of the open-ended, free responses, in which the top three most frequent themes include “Skill Development” (n=48, 67%), “Teamwork” (n=26, 36%), and “Role Rotation” (n=23, 32%), reflecting students' appreciation for comprehensive and collaborative CS practice and acquisition. Other identified themes include “Equity Fairness” (25%), “Facilitation Organization” (n=18, 15%), “Engagement” (n=11, 15%), “Constructive Criticism” (n=10, 14%), “Feedback/Reflection” (n=10, 14%) responses, and “Clinical Preparation” (n=10, 14%) responses.

**Figure 3:**
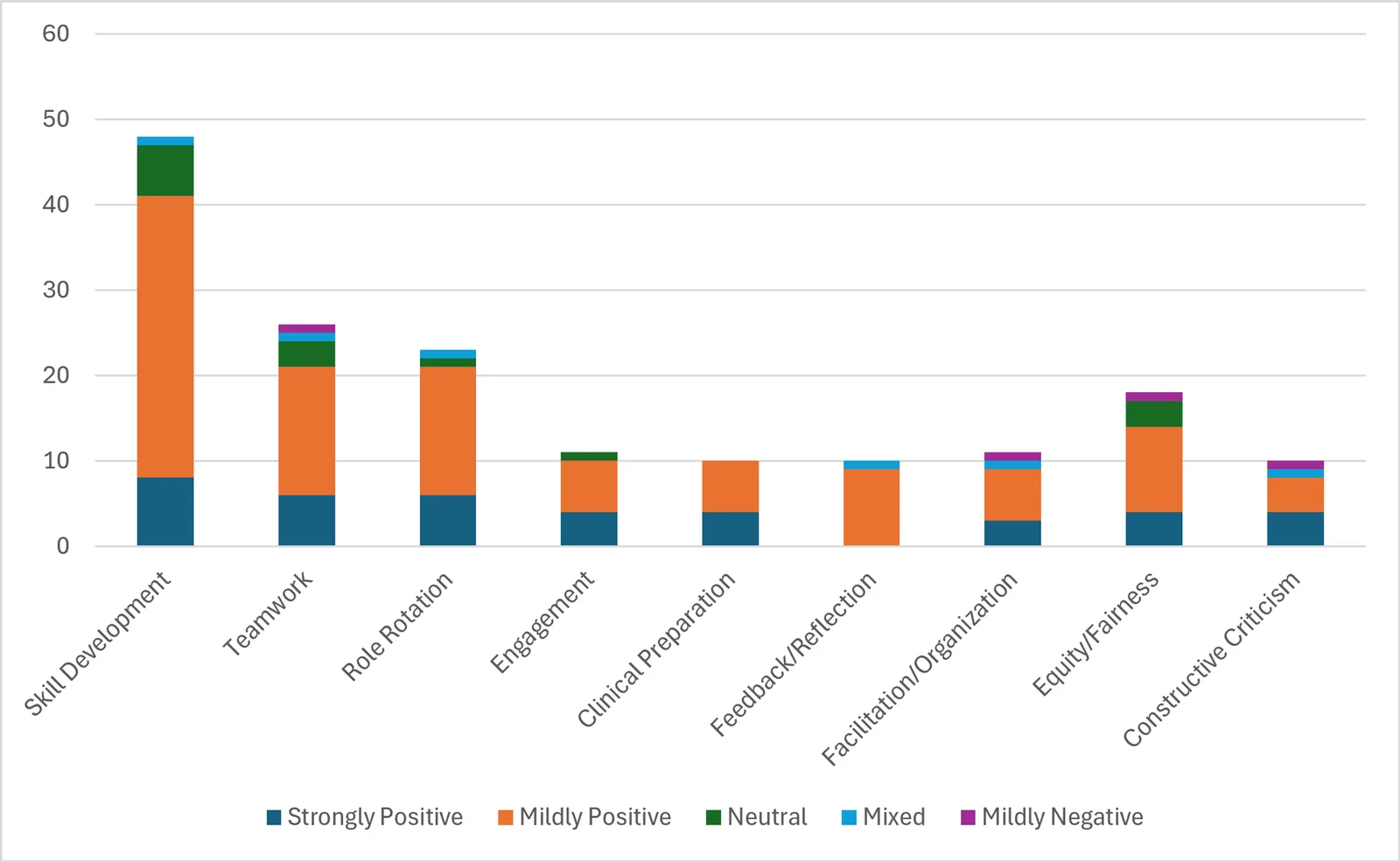
Sentiment Breakdown by Theme

Figure 3 shows the sentiment breakdown by theme. Positive sentiment (mild and strong) was mostly identified among the top three most frequent themes. Eight (11%) strongly positive sentiments and 33 (46%) mildly positive sentiments were identified for “Skill Development” themes, and six (8%) strongly positive sentiments and 15 (21%) mildly positive sentiments were identified for each of the “Teamwork” and “Role Rotation” themes.

**Figure 4:**
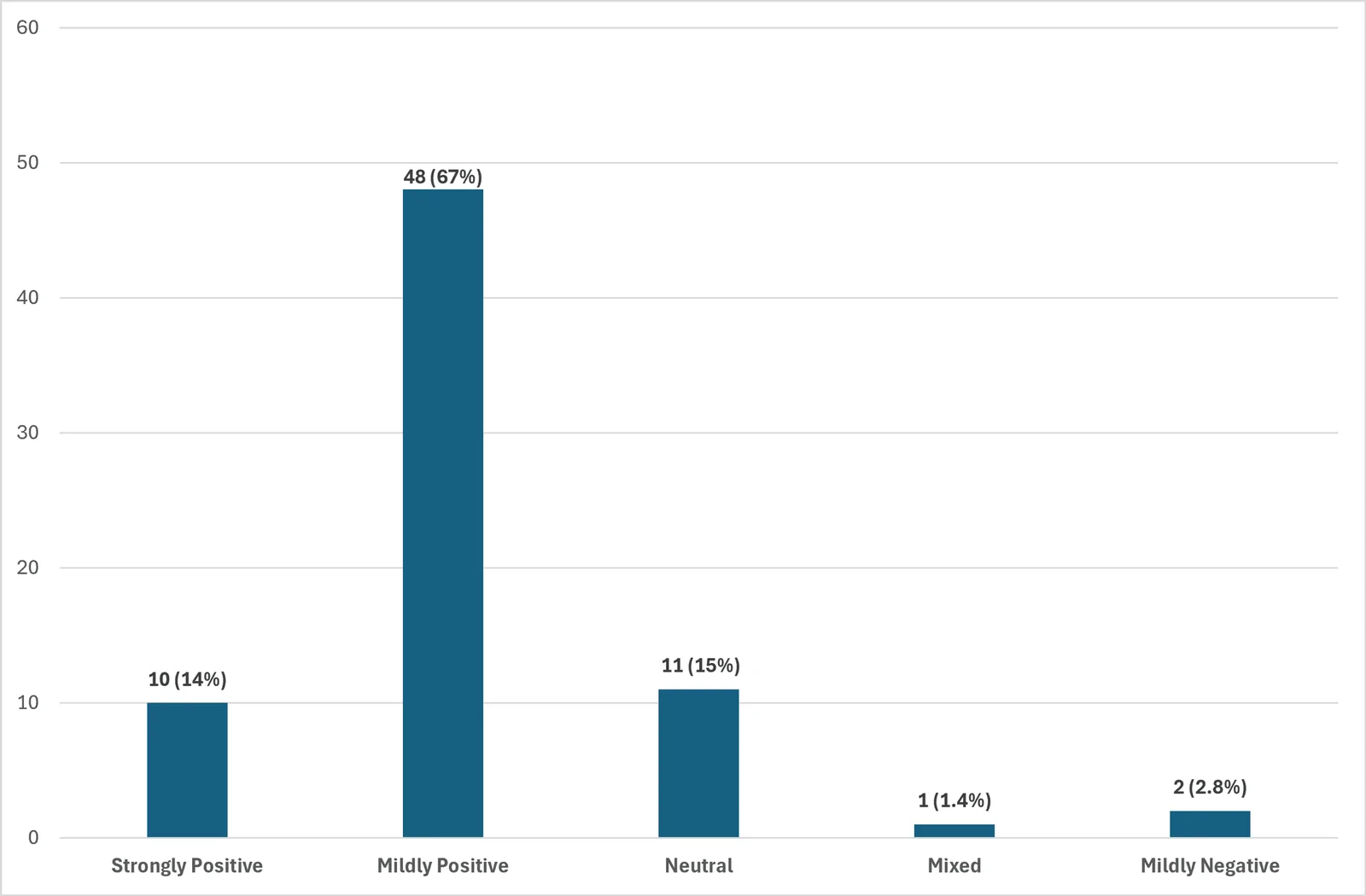
Distribution of Sentiment Count in the 72 Responses

The findings of the sentiment analysis showed 58 (80.6%) of the 72 responses carried “Positive” sentiment, with 10 (14%) responses classified as “Strongly Positive” and 48 (67%) responses as “Mildly Positive”. Other sentiments include 11 (15%) responses classified as “Neutral”, two (2.8%) as “Mildly Negative”, and one (1.4%) as “Mixed”. No response was classified as “Strongly Negative”. Figure 4 shows the sentiment count distribution.

**Figure 5:**
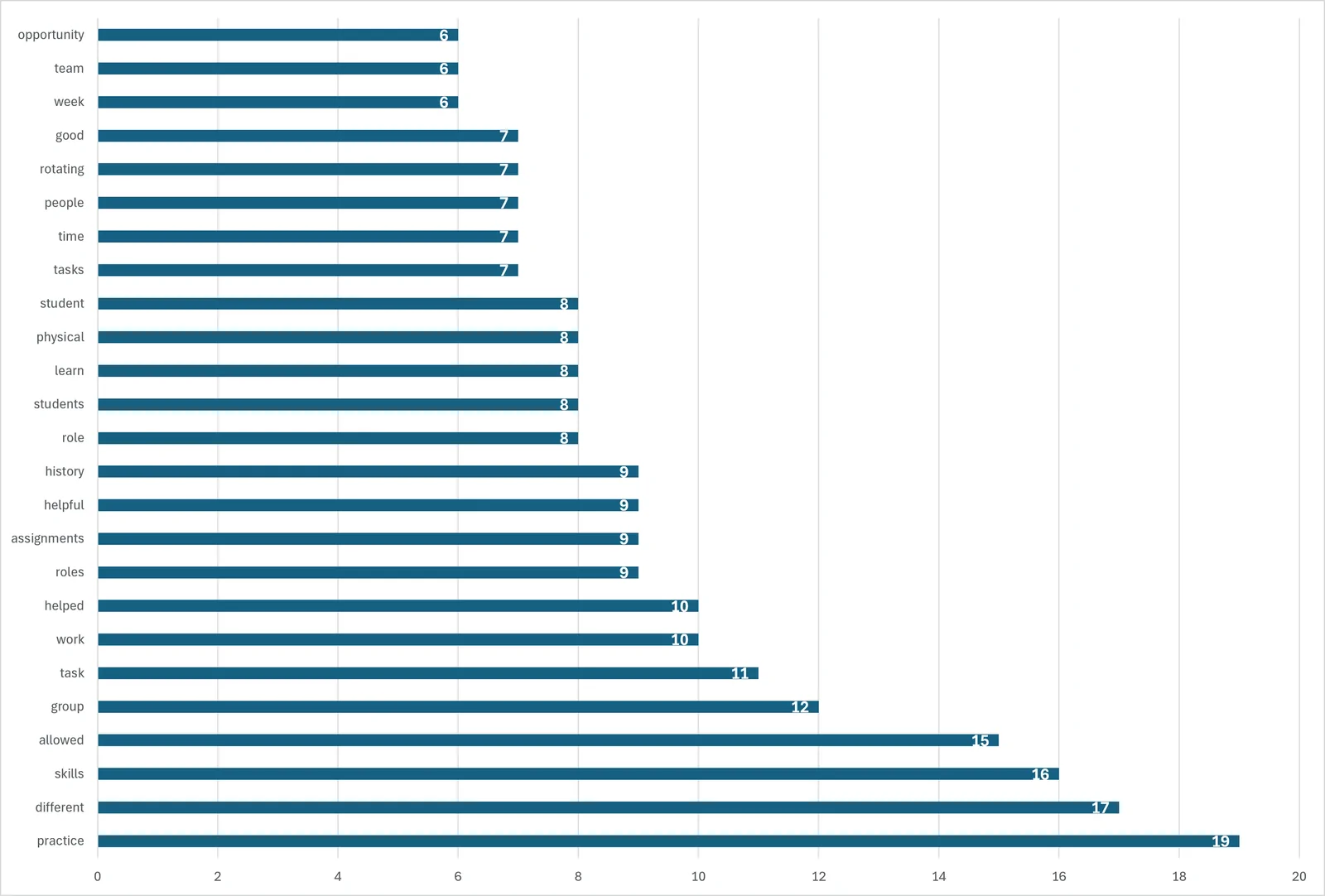
The Top 25 Frequent Words Mentioned in the Free Responses

The content analysis identified five most frequently mentioned words in the responses: “practice" (n=19, 26%), “different” (n=17, 24%), "skills" (n=16, 22%), “allowed" (n=15, 21%), and "group" (n=12, 17%), confirming that students frame the TBS-SPE format primarily in terms of diverse experiential learning and collaborative benefit. Figure 5 shows the 25 words most frequently mentioned in the free responses.

**Figure 6:**
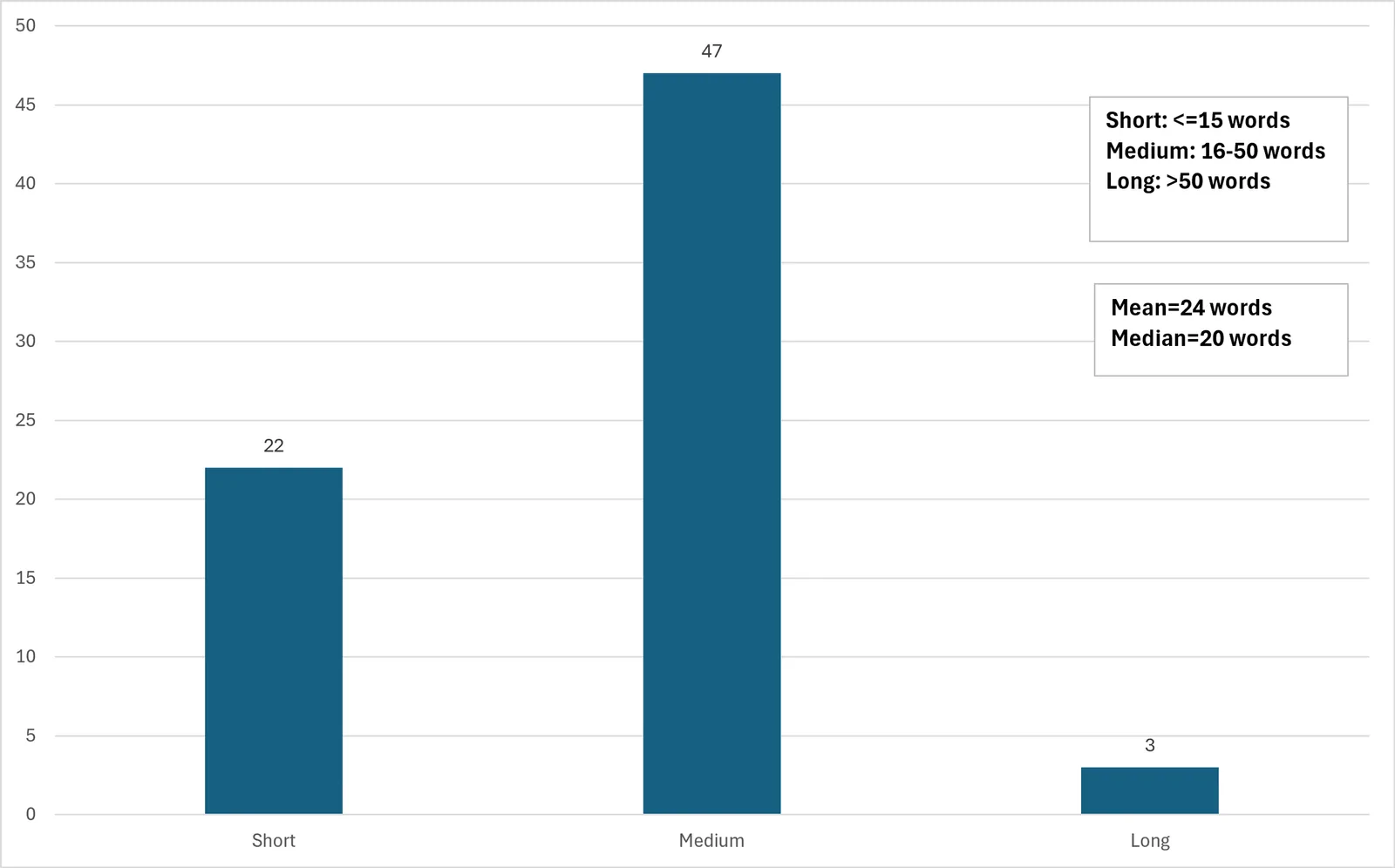
Distribution of the Free Responses by Length

Figure 6 shows that 47 (65%) of the responses were classified as “Medium” length responses, 22 (31%) as short, and three (4%) as long. The mean and median of the response length by word were 24 and 20, respectively.

## Discussion

Learning in CS courses goes beyond motor skill observation and practice and encompasses the application of medical knowledge into the clinical reasoning process that guides the process of history taking, physical examination, formulation of the differential diagnosis, and discussion of the management plan. In addition, interpersonal and verbal and nonverbal communication skills are also at the heart of CS learning. An ideal CS session would engage all students in a small group in different roles addressing these knowledge, skills, and attitude components. The faculty who usually facilitate the learning in CS small groups come from various specialties and encompass various approaches in addressing the weekly CS session content. This, coupled with a lack of a standardized session structure, has contributed to variability in student engagement, learning, and assessment across different CS student groups assigned to different faculty. Our TBS-SPE approach has effectively contributed to optimization of CS learning and minimization of variability among student groups.

Students provided the two faculty members who initiated the TBS-SPE approach with very positive feedback and rated them highly in the faculty evaluation for how the sessions were organized and conducted. Later, when the approach was shared and was recommended to be used by all CS faculty, more faculty started to receive positive feedback on the use of TBS-SPE in their sessions. Those individual positive sentiments are now supported and validated by the official results of our study. The students felt that they were constantly involved in each session even when they were just observing peers performing skills, while they waited for their turn. This setting allowed for peer-to-peer learning. Since their assigned roles were only revealed at the time of the session, students had to come to the session prepared to take on any of the roles preplanned by faculty.

Students from both POM-2 and POM-3 courses have equally rated the approach highly, as proved by the score mean and the non-significant difference in the high ratings from both cohorts over two academic years. This consistent and homogeneous high rating of the TBS-SPE approach across different CS courses indicates the uniform acceptance and success of the approach as a valuable CS learning experience.

Most of the students provided positive sentiments in their free responses. The top five themes framing these sentiments are: “Skill Development” being the most frequent, followed by “Teamwork”, “Role Rotation”, “Equity/Fairness”, and “Facilitation Organization”, respectively. Students consistently described the rotating roles setting of the TBS-SPE as a way to build a broad and balanced clinical skillset. Many emphasized that it pushed them to identify areas of improvement rather than relying on strength alone. Some quotes from the free responses by students related to these sentiments include:

“Allowed us to practice the respective tasks and thereby solidify the skills necessary therein.”

*“The rotation of assignments allowed us to work on our weak areas even if we would have rather focused on our areas of strength.”* 

The three (4.1%) responses that were classified as mixed-mildly negative were mainly focused on the group size, the identification of skill areas students need to have more exposure to, and adding engaging activities for students who are not actively participating. The majority (65%) of the responses were of medium length, reflecting the contextually balanced, reasonable, and actionable nature of the communicated opinions. The content analysis showed the five most frequent words mentioned were: "Practice", "Skills", “Different", “Group”, and "Allowed". This indicates that students felt the structure of the TBS-SPE approach gave them permission and/or opportunities to practice various skills in a group setting through tasks they might otherwise have avoided.

In our CS courses, faculty meet with the students three times during the course, at the beginning, halfway through, and before the course conclusion. During these meetings, faculty discuss with the students their individualized learning plan that addresses their strengths and areas for improvement. This information is highly considered by the faculty when they assign students to different roles that would expose them to practice the skills they need to improve on.

The group size ranges from seven to eight students per group, which is an ideal number that allows us to cover all tasks planned and assigned to students in the weekly sessions. The students who are not actively participating and wait for their turns actually observe and learn from their peers who are actively practicing the assigned roles. All students are invited to participate in peer feedback at the end of each round of history taking, physical examination, and encounter closure. The tasks of formulating the differential diagnosis and proposing a management plan are assigned to individual students who lead the related discussions and engage the whole group.

While our TBS-SPE approach has been instrumental in minimizing variability across different groups and enhancing acquisition of knowledge and skills in the CS course, it is not the only effort course directors have dedicated to that purpose. In addition, to boost the effectiveness of TBS-SPE, they hold several faculty development sessions with the CS facilitators, as our faculty members represent a broad spectrum of specialties including general surgeons, orthopaedic surgeons, internists, pathologists, obstetricians and gynecologists, and pediatricians among others. It is important to us that facilitators are educated on the implementation of the structured CS sessions and feel confident in delivering the sessions, since the topics being discussed may not be within their specialty scope. In these faculty development sessions, physical examination maneuvers are reviewed with faculty prior to reviewing them with the students; this has allowed us to enhance our efforts in minimizing variability across CS groups.

Our TBS-SPE approach echoes national efforts to enhance CS learning through integration of basic and clinical science content in the clinical reasoning process that guides the SP encounter. Kelekar and Afonso studied the impact of a pre-clerkship CS clinical reasoning curriculum on the outcomes of the final Objective Structured Clinical Examination [13]. They concluded that the emphasis of clinical reasoning in the CS courses has effectively guided students’ performance and enhanced their critical thinking and interpretational skills needed to formulate a relevant differential diagnosis. Uchida et al. reported that the majority of medical schools in the United States integrate physical examination with history taking through a hypothesis-driven approach that is based on relevantly interacting basic science correlations [14]. 

Our weekly TBS-SPE sessions include demonstration of skills by the faculty, facilitating small group CS learning which is preceded by faculty explanation of the clinical significance and rationale to apply those skills in advance [6-9]. While our TBS-SPE sessions do not explicitly include “near peer teaching”, it indirectly incorporates related elements through faculty-facilitated student-led clinical reasoning discussions and the interchangeable skill-practicing-feedback experiences. Hari et al. concluded that practical skills curricula should integrate and make the most of the values of both faculty and near-peer inputs [15]. One of the major factors that has contributed to the effectiveness of TBS-SPE is the structured feedback part of the process. SPs provide feedback on how they felt during the encounter in areas related to student performance in interpersonal and communication skills. Faculty and peers provide feedback on students' practicing skills, comparing the performance to the standard they learned from faculty demonstrations and other related resources, e.g., educational videos. Informed expectations and formative feedback on student performance are key elements in learning CS [16]. The weekly session material also includes a detailed session guide that explains the learning expectations of the session, and all provided logistics and learning resources.

Another significant factor of the TBS-SPE is the enhancement of active learning through the application of knowledge and skills this approach has allowed. From history taking, physical examination, discussing the management plan, closing the encounter, to orally presenting the case findings, students were experiencing settings that required application of clinical reasoning and other skills in a fashion that mimics their future interactions with real patients [17,18].

Finally, our rotating multitask approach has not only ensured equal opportunity learning where all students participate in different roles in one session, but it also enhanced the acquisition of knowledge and skills needed to practice the individual components of the complex task of patient consultation; an undertaking they will practice in residency and beyond [19].

 Challenges and limitations

Even though the TBS-SPE approach has revealed meaningful perceived effectiveness by students in enhancing CS acquisition, there are some important challenges and limitations which warrant recognition. Firstly, this study was conducted at a single allopathic medical school. Therefore, the data obtained from this study may limit the generalizability of findings given the variability of other medical schools with respect to the composition of CS courses, availability of resources, faculty make-up, and ratios of students to faculty. However, the principle of structuring the small group CS session on rotating tasks scaffolding is a generic concept that could be adapted into the various settings of CS courses throughout different institutions.

Second, the evaluation of TBS-SPE effectiveness is based primarily on student self-reported perceptions using a Likert-scale rating and open-ended responses to questions. These measures represent opinion-based indirect assessments of student learning that are mostly subjective depictions of student satisfaction and perceived learning. Like other perception-based outcomes, free-response opinions may be associated with some constraints, e.g., expectation bias, response calibration, and social desirability biases. To mitigate these risks, the survey was anonymous and privately self-administered, with study-related questions embedded within the broader end-of-course evaluation. Additionally, the use of AI could also be considered a methodological limitation related to reproducibility and classification validity that depend on the prompting and human verification processes. To address this, we carefully reviewed the consistency of the prompt outcomes and the categories’ definitions, and we cross-referenced the results with the original response texts. Nevertheless, future studies should incorporate direct objective assessments of CS learning through linking session learning expectations to course objectives in the blueprints of related CS assessments. Reviewing such measures may allow a more direct and explicit evaluation of the effect of TBS-SPE on skill acquisition and retention. For this purpose, the authors are planning to utilize the course curriculum map to integrate the connections across different-level TBS-SPE-related objectives into CS assessment blueprints and analyze the assessment results accordingly.

Among other analytical findings is the survey response rate for the open-ended questions, which shows only 72 of 225 students (32%) providing qualitative responses. Nevertheless, the response rate for the related five-point Likert scale question was 100%, and the means for both courses involved were very positive (POM-2: 4.27 and POM-3: 4.3). This could be considered a prediction suggesting that even those who did not answer the related open-ended question could be satisfied and valued the TBS-SPE approach.

Finally, the execution of TBS-SPE is heavily dependent on having sufficient faculty development and support. Our CS faculty belong to a variety of specialties; thus, consistent and frequent faculty training was required for the success of TBS-SPE. In some institutions with limited faculty development resources, tremendous variability in the implementation of this approach could be expected. 

## Conclusions

The TBS-SPE approach is designed to enhance the delivery of CS during the pre-clerkship medical education training. When tasks are assigned and rotated during the weekly SP encounter sessions, students are provided with a comprehensive, equitable, and structured engagement in small group settings. The results from this study show high Likert-scale scores across two cohorts of medical students in two consecutive academic years, combined with positive student reflections on skill development, teamwork, and role rotation. These results demonstrate that students valued the TBS-SPE teaching method and appreciated its positive impact on their CS learning. 

The innovative approach described in this study provides a solution to effectively managing variability in the CS teaching and learning process. By providing access to a standardized rotational schedule that is flexible and comprehensive, faculty can quickly adopt this approach regardless of specialty while still preserving their own facilitation style within the assigned student small groups. Based on the findings of this study, it is recommended that CS course directors and faculty consider the adoption of the concept of TBS-SPE as a standardized CS session framework. Future research should aim to include objectively measured competency-based outcomes that allow for longitudinal evaluations. 
